# Policy Series: COVID-19: How It Shaped Nursing Home Care and Elder Justice

**DOI:** 10.1093/geroni/igab046.1499

**Published:** 2021-12-17

**Authors:** Brian Lindberg

## Abstract

This session will provide updates on how the pandemic led to horrific situations in long-term care facilities and how the pandemic influenced major federal efforts to address elder abuse, neglect, and exploitation.

